# The potential of bacterial anti-phagocytic proteins in suppressing the clearance of extracellular vesicles mediated by host phagocytosis

**DOI:** 10.3389/fimmu.2024.1418061

**Published:** 2024-06-06

**Authors:** Jiacong Sun, Congcong Chen, Pengpeng Pan, Keyi Zhang, Jinrui Xu, Cheng Chen

**Affiliations:** ^1^ School of Life Sciences, Tianjin University, Tianjin, China; ^2^ Key Laboratory of Anti-inflammatory and Immune Medicine, Ministry of Education, Institute of Clinical Pharmacology, Anhui Medical University, Hefei, China; ^3^ School of Life Sciences, Ningxia University, Yinchuan, China; ^4^ Key Laboratory of Ministry of Education for Conservation and Utilization of Special Biological Resources in the Western, Ningxia University, Yinchuan, China

**Keywords:** extracellular vesicles, clearance, phagocytosis, bacterial anti-phagocytic proteins, targeted therapy, tumor deactivation, synergistic effect

## Abstract

Extracellular vesicles (EVs), characterized by low immunogenicity, high biocompatibility and targeting specificity along with excellent blood-brain barrier permeability, are increasingly recognized as promising drug delivery vehicles for treating a variety of diseases, such as cancer, inflammation and viral infection. However, recent findings demonstrate that the intracellular delivery efficiency of EVs fall short of expectations due to phagocytic clearance mediated by the host mononuclear phagocyte system through Fcγ receptors, complement receptors as well as non-opsonic phagocytic receptors. In this text, we investigate a range of bacterial virulence proteins that antagonize host phagocytic machinery, aiming to explore their potential in engineering EVs to counteract phagocytosis. Special emphasis is placed on IdeS secreted by *Group A Streptococcus* and ImpA secreted by *Pseudomonas aeruginosa*, as they not only counteract phagocytosis but also bind to highly upregulated surface biomarkers α_V_β_3_ on cancer cells or cleave the tumor growth and metastasis-promoting factor CD44, respectively. This suggests that bacterial anti-phagocytic proteins, after decorated onto EVs using pre-loading or post-loading strategies, can not only improve EV-based drug delivery efficiency by evading host phagocytosis and thus achieve better therapeutic outcomes but also further enable an innovative synergistic EV-based cancer therapy approach by integrating both phagocytosis antagonism and cancer targeting or deactivation.

## 1 Introduction

Extracellular vesicles (EVs) are a versatile group of cell-secreted, membrane-enveloped spherical particles present in body fluids (1). Ranging from 30 to 5,000 nm in diameter, EVs are further classified into three types based on their size and origins: exosomes, microvesicles, and apoptotic bodies. Exosomes, naturally vesicles with a diameter of 30 to 150 nm, are derived from endosomal pathway, followed by active release into extracellular environment through exocytosis. In contrast, microvesicles, which have a diameter of 100 to 1,000 nm, are originated from the plasma membrane through budding process. And apoptotic bodies, subcellular particles which have significantly larger diameters, typically ranging from 1,000 to 5,000 nm, are released following programmed cell death (2, 3).

Currently, EVs have been proved to work as pivotal mediators in intercellular communication by transmitting signals from donor cells to recipient cells through specific ligands and receptors on their surfaces (3). EVs have accordingly evolved a range of unique advantages to support their regulatory role in such physiological processes as inflammation, tumorigenesis, angiogenesis, and cell fate control (1, 4). Firstly, all three types of EVs are capable of transporting highly diversified molecules including proteins, RNA, nuclear components, organelles, and other metabolites as effectors to target cells (3, 5). Secondly, EVs are highly stable and biocompatible due to their lipid bilayer membrane shell which shield the cargo they enclosed from degradation, promote fusion of EVs with recipient cells and facilitate EVs to easily traverse the human blood-brain barrier (4). Thirdly, EVs sourced from different cells have a notable variation in surface composition and hence can bind to different recipient cells through surface-specific molecules, imparting EVs natural targeting specificity (6). Due to these intrinsic properties, EVs are currently widely used as drug delivery systems for therapeutic purpose in conditions such as cancer, inflammation, and viral infections (7–9). For example, nanovesicles carrying a fusion protein composed of SIRPα and PD-1 variants on their surface effectively induce anti-tumor immunity of T cells and macrophages (10). Macrophage-derived microvesicles loaded with dexamethasone can suppress renal inflammation and fibrosis (11). EVs expressing ACE2 demonstrate 60 to 80 times greater efficacy in preventing SARS-CoV-2 infection compared to the purely soluble ACE2 extracellular domain (9).

In this perspective, we will review current progress on the clearance mechanism exerted by host mononuclear phagocyte system towards EVs. Next, by carefully inspecting bacteria-host interplay in terms of macrophage phagocytosis antagonization, we will propose the potential application of bacterial anti-phagocytic proteins in engineering and protecting EVs from host phagocytosis. Notably, for those proteins such as IdeS from *Group A Streptococcus*(GAS) and ImpA from *Pseudomonas aeruginosa*, an innovative synergistic EV-based strategy aimed for more effective cancer therapy is further conceived based on their additional functions concerning tumor targeting or deactivation.

## 2 Fast clearance of EVs by mononuclear phagocyte system

As a crucial mediator in intercellular communication, EVs bud from internal or plasma membranes of almost all cells, encapsulate cargoes for transportation into extracellular space and are adorned with a diverse array of molecules including lipids, polysaccharides, and proteins on outer surfaces (12, 13). While these molecules facilitate targeted delivery of cargo-encapsulated EVs to receptor cells, they are meanwhile effectively recognized by various receptors expressed on phagocyte surface, such as immunoglobulin Fc receptors, complement receptors, and scavenger receptors. Due to this, phagocytosis will be inevitably triggered in host phagocytes, resulting in fast clearance of EVs (4, 14). In a study performed by Willekens, 80% of the radiolabeled red blood cell-derived EVs were cleared from bloodstream in just 5 minutes after they were injected into the veins of mice (15). Similarly, the plasma-derived EVs rapidly disappear with a half-life of approximately 7 minutes in a macrophage-depleted mouse model (16). Meanwhile, Watson and colleagues demonstrated that blocking the class A scavenger receptor (SR-A) with dextran sulfate significantly decreased the *in vivo* liver uptake of EVs in mice (17). To sum up, for EVs acting as drugs or delivery tools, the rapid uptake of them by host mononuclear phagocyte system (MPS) leads to a great reduction in their quantity and finally impaired clinical efficacy.

**Figure 1:**
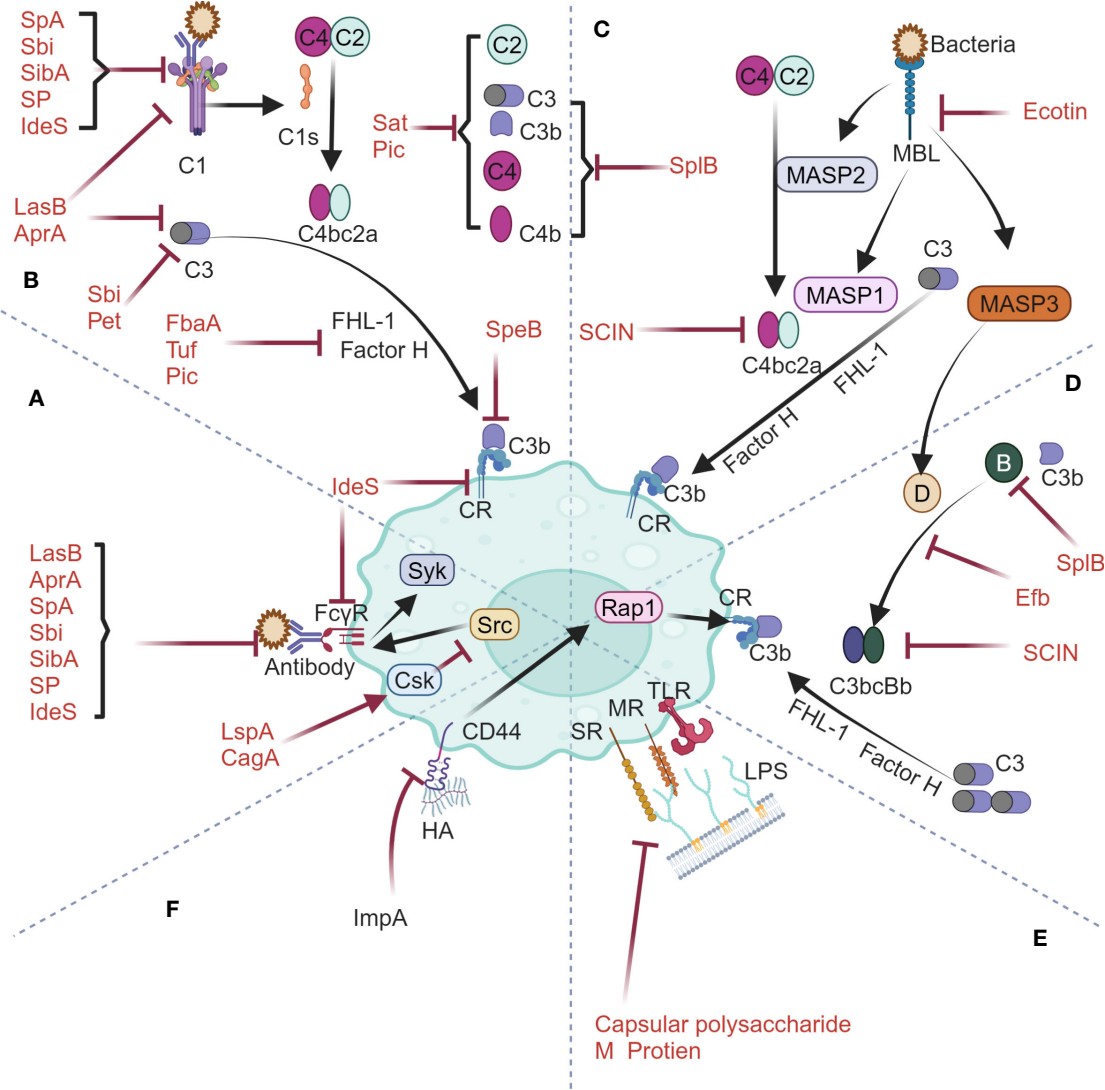
Phagocytosis machinery by host mononuclear phagocytic system and its interplay with bacterial anti-phagocytic proteins spanning Fcγ receptor-mediated pathway **(A)**, classical complement pathway **(B)**, mannose-binding lectin pathway **(C)**, alternative complement pathway **(D)**, non-opsonic phagocytic receptor-mediated pathway **(E)** and CD44 phagocytic receptor-mediated pathway **(F)**. The figures were created using biorender.com.

Phagocytosis relies on recognition of various receptors on phagocyte surfaces to initiate downstream signaling cascades. Phagocytic receptors are categorized into opsonic and non-opsonic receptors. Opsonic receptors consist of Fc receptors and complement receptors, which mediate internalization of EVs by recognizing immunoglobulins (Igs) and complement proteins (14). For Igs, they firstly bind to the Fcγ receptors on extracellular surface of phagocyte and then trigger intracellular phosphorylation of tyrosine residues within the immunoreceptor tyrosine-based activation motif (ITAM) via Src family kinases. The phosphorylated ITAM domain will further recruit the tyrosine kinase Syk and thereby starts phagocytosis (18, 19) (**Figure 1A**). For complement proteins, they are found to regulate phagocytosis via three pathways, namely classical, lectin, and alternative pathways. The classical pathway is initiated by activation of C1 upon the binding of IgG and IgM, followed by cleavage of C4 and C2 and finally formation of the C3 convertase C4bC2a (**Figure 1B**). The lectin pathway involves such soluble carbohydrate-binding lectins as mannose-binding lectin (MBL), and activating MBL-associated serine proteases (MASPs), with MASP1 directly cleaving C3 and MASP2 activating C4 and C2 to generate the C3 convertase C4bC2a (**Figure 1C**). The alternative pathway is activated when C3b binds to factor B, which is then cleaved by factor D into Bb, forming the C3 convertase C3bBb (**Figure 1D**). The C3 convertases (including C4bC2a and C3bBb) from either pathway will cleave C3 to generate large amounts of C3b and C3a. Complement regulatory protein FHL-1 then mediates the cleavage of cell surface-bound C3b into iC3b, which, through interaction with complement receptors (CR), finally starts phagocytosis process (20, 21) (**Figures 1B-D**). As for non-opsonic phagocytosis process, a range of non-opsonic receptors such as mannose receptor (MR), scavenger receptor (SR) and Toll-like receptors (TLR) are utilized to recognize various ligands, followed by cellular engulfment and clearance of these targets (22–24). Basically, MR from macrophages mainly mediates the engulfment and uptake of pathogens by recognizing specific glycosylated structures such as mannose, fucose, and N-acetylglucosamine present on the surface of pathogens (25). The scavenger receptor (SR) with broad ligand specificity mainly mediates phagocytosis by binding to various ligands such as lipoteichoic acid (LTA), lipopolysaccharide (LPS), CpG DNA, and other pathogen-associated molecular patterns (PAMPs) to facilitate cellular engulfment (26) (**Figure 1E**).

## 3 Engineering EVs with host anti-phagocytic molecules

To inhibit the clearance of EVs by phagocyte and hence achieve more efficient targeted delivery of drug molecules, researchers have engineered EVs by integrating anti-phagocytic molecules onto their surfaces, in hopes of enabling EVs to counteract phagocytosis. Currently, a bunch of host molecules such as CD47, CD24, CD44, CD31, β2M and PD-L1 serve as candidates that may exert an anti-phagocytic role for EVs, among which CD47, the ligand for SIRPα is the most researched one (27). Studies have shown that CD47-SIRPα binding can induce tyrosine phosphorylation within the ITIM motif of SIRPα, followed by the recruitment of SHP-1. This process inhibits phagocytosis mediated by Fcγ receptors, complement receptors, or low-density lipoprotein receptor-related protein 1 (LRP1) (28, 29). The CD47-SIRPα interaction is thus thought to endow EVs with a “don’t eat me” signal and regarded as one promising approach by which cells evade immune surveillance (27). In an earlier work performed by Kamerkar and colleagues, when exosomes derived from normal fibroblast-like mesenchymal cells were engineered with CD47 and used to carry siRNA/shRNA to pancreatic cancer cells with KRAS mutations, fairly limited exosome clearance by phagocyte and meanwhile enhanced accumulation of microRNA were successfully observed (30). Progress have been made in utilizing CD47 to antagonize phagocytosis. However, as a multifunctional molecule, CD47 has a much broader role in physiological processes. For example, CD47 also mediates the TSP1-CD47 signaling cascade, participates in cell cycle regulation, and possesses the ability to induce cellular senescence and death (31, 32). Therefore, EVs carrying CD47, when used in delivery process to evade phagocyte, may potentially interfere with normal functions of the host cells.

## 4 Bacterial proteins inhibit phagocytosis

In nature, bacteria represent a major group of pathogens that coevolve with host immune system. Being the most basic defense mechanism in innate immunity, phagocytosis discussed in this text has complex interplay with different bacteria (23, 33) (**Figure 1**). It’s reasonably anticipated that specific proteins shall be produced by bacteria to interfere with phagocytic process for preventing them from being eliminated (34). Consequently, these diversified proteins objectively become candidates for engineering EVs to counteract host phagocytosis.

### 4.1 Anti-phagocytic proteins expressed by gram-positive bacteria

GAS encoded two important cysteine proteases in terms of antagonizing host phagocytosis (**Figures 1A, B**). One is IdeS, a specific proteinase which can not only cleave the hinge region of IgG to generate one F(ab)_2_ fragment and two 1/2Fc fragments but also bind competitively to FcγRIII and complement receptor CR3, hence being capable of inhibiting phagocytosis mediated by human Fcγ receptors and complement receptors (35, 36) (**Figures 1A, B**). The other is SpeB, a nonspecific proteinase which can cleave various host proteins including C3b and hinder complement activation, thereby disrupting the host’s regulatory phagocytic response to bacteria (37). Apart from this, the fibrinogen-binding protein FbaA expressed by GAS can bind to the human complement regulatory proteins factor H and FHL-1, thereby blocking the deposition of C3b onto the nuclear surface (38) (**Figures 1A, B**).

*S. aureus* encoded four virulent proteins related to antagonizing host phagocytosis. The serine protease (SP) can cleave IgG and IgM, thereby inhibiting phagocytosis mediated by Fcγ receptors and complement receptors (39) (**Figures 1A, B**). The secreted SpA protein and Sbi protein can bind to the Fc portion of IgG while Sbi also binds to C3, thereby interfering with the activation of Fcγ receptors and complement pathway (40–42) (**Figures 1A, B**). The Staphylococcal complement inhibitor protein (SCIN), which targets the C3 convertase, blocks the complement pathway through specific interactions with C4b2a and C3bBb (43) (**Figures 1C, D**). Additionally, the extracellular fibrinogen-binding protein (Efb) blocks the formation of the C3 convertase by inhibiting the binding of factor B to C3b (44) (**Figure 1D**). SplB can inhibit the three complement pathways by degrading complement molecules such as C3, C3b, C4, C4b, and factor B (45) (**Figures 1B-D**).

### 4.2 Anti-phagocytic proteins expressed by gram-negative bacteria

*P. aeruginosa* encoded four virulent factors concerning the antagonization of host phagocytosis. Elastase LasB and alkaline protease AprA can cleave Igs and complement C1a and C3 and matrix metalloprotease ImpA can cleave and disrupt the signaling cascade of macrophage surface phagocytic receptor CD44, hence inhibiting complement receptors-mediated phagocytosis (46–48) (**Figures 1A, B, F**). The Tuf factor on the surface of *P. aeruginosa* can bind to the complement regulatory factor H to inactivate C3b, thus evading complement attack of the host (49) (**Figure 1B**). Apart from these factors, LspA protein from *H. ducreyi* and CagA protein from *H. pylori* can effectively inhibit the activity of Src family protein tyrosine kinases by activating the catalytic function of C-terminal Src kinase (Csk), therefore impeding phagocytosis mediated by Fcγ receptors (50) (**Figure 1A**).

*E. coli* encodes four virulence proteins involved in the inhibition of complement-mediated phagocytosis, among which, the staphylococcal acetyltransferase Sat along with the toxins Pet and Pic can inhibit three complement pathways by cleaving various complement molecules (**Figures 1B-D**). In particular, Pic can also synergize with the complement regulator factor H to inactivate C3b (51–53) (**Figures 1B**). Apart from this, the Ecotin protein can inhibit the lectin pathway by inactivating MASP-1 and MASP-2, and meanwhile suppress the activation of the alternative pathway through inhibition of the activation factor D of MASP-3 (54) (**Figure 1C, D**).

The bacterial protein listed above mainly target the opsonic-phagocytosis pathway (**Figure 1**). The reason for this is that bacteria normally cloak their membrane surfaces with extracellular polysaccharides such as capsules and alginates, which effectively cover the ligands and impede the recognition of ligands by non-opsonic phagocytic receptors on macrophages surface (55, 56) (**Figure 1E**). However, *S. pyogenes* evolves a specific mechanism of counteracting host non-opsonic phagocytosis activity during which process the M protein on membrane surface inhibits recognition of the bacteria itself by targeting host macrophage SR-A receptors (57) (**Figure 1E**). In summary, both Gram-positive bacteria and Gram-negative bacteria can secrete specific virulence proteins, which, by means of binding to or cleaving critical molecules involved in phagocytic mechanism, effectively evade opsonic phagocytosis (58).

## 5 Construction of an innovative synergistic EV-based cancer therapy approach

**Figure 2:**
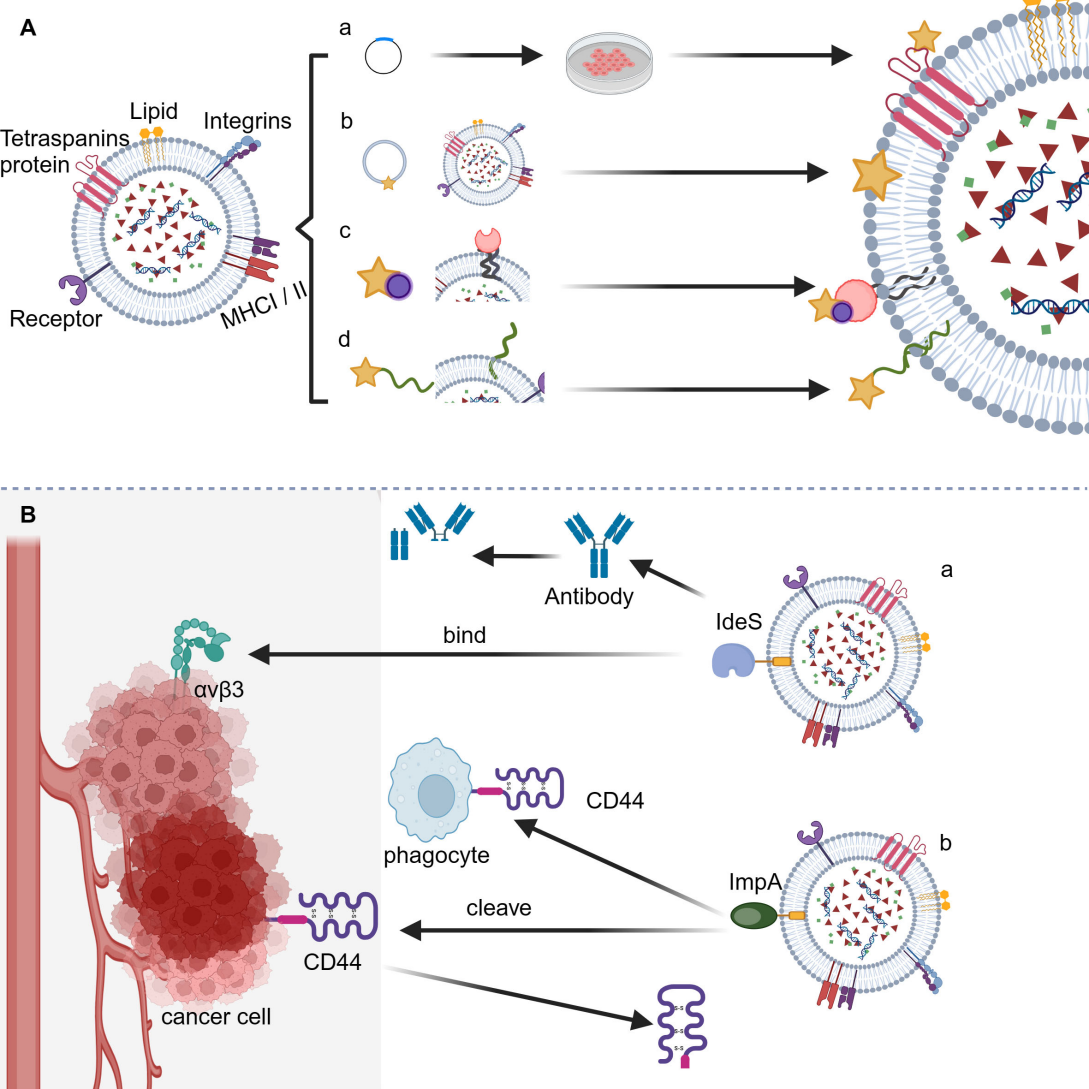
Application of bacterial anti-phagocytic proteins for counteracting host phagocytosis. **(A)** The loading strategies for bacterial anti-phagocytic proteins onto EVs. (a) Genetic modification; (b) Direct membrane fusion; (c) Biotin-streptavidin interaction; (d) PEG surface labeling. **(B)** The sheme of synergistic EV-based cancer therapy approach. (a) IdeS-αVβ3 interaction facilitates tumor targeting; (b) Potential cleavage of CD44 by ImpA enables tumor deactivation.

Targeted therapy, an innovation revolutionizing cancer treatment based on profound analysis of biological disparities between tumor and normal tissues, aims to eliminate cancer cells selectively by identifying and regarding receptors or signaling pathways overexpressed exclusively in tumor cells as drug targets while minimizing collateral damage to healthy cells (59, 60). Thus, drug-loaded nanocarriers that possess high targeting specificity or deactivation properties towards tumor cells should significantly enhance treatment outcomes, as they increase drug concentration within tumor tissues by precise drug delivery or exert tumor deactivation effects, respectively, ultimately improving the effectiveness of targeted therapy (61, 62). In this context, it is discovered that EVs modified with bacterial proteins IdeS and ImpA may possess the above function. To achieve this, the loading of bacterial proteins onto the surface of EVs is firstly required. Currently, two primary approaches, namely pre-loading and post-loading strategies, have been developed (63). The former mainly involves transfecting parent cells with vectors encoding the desired proteins that are not naturally present or vectors expressing recombinants by fusing desired proteins to such host proteins as CD63, CD9, CD81, or LAMP2b. The latter, on the other hand, allows the loading of target proteins onto the surface of EVs mainly by biochemical techniques such as biotin-streptavidin interaction, lipid membrane fusion, and PEG surface labeling (64, 65) (**Figure 2A**). We reasonably believed that engineered EVs with bacterial proteins IdeS and ImpA loaded onto their surface can be achieved using the above techniques.

### 5.1 Tumor targeting through IdeS-α_V_β_3_ interaction

Integrin signal transduction plays a crucial role in mediating the adhesion of epithelial cells, thus regulating various biological behaviors such as migration, invasion, proliferation, and tumorigenesis (66). A number of integrins are found to have significant differences in expression levels between tumor and normal tissues and hence provide potential targets for cancer therapy. Among various integrins, the α_V_β_3_ integrin has significantly upregulated expression level in cancers such as melanoma, breast cancer, prostate cancer, pancreatic cancer, ovarian cancer, cervical cancer, and glioblastoma compared to that in normal epithelial cells and is thus identified as an effective marker and drug target for cancer (67, 68). Based on the fact that integrin α_V_β_3_ can specifically bind to the Arg-Gly-Asp (RGD) motif present in various extracellular matrix (ECM) ligands, researchers have developed several targeted drugs carrying RGD motif, among which cyclic RGD peptide Cilengitide has shown promising prospects in clinical trials targeting a range of cancers including lung cancer, prostate cancer, and glioblastoma (66, 69, 70). The IdeS from GAS contains the RGD motif and can effectively bind to integrins α_V_β_3_ and α_IIb_β_3_, enabling the potential application of IdeS for effective tumor targeting. Notably, compared to IdeS of M1 serotype, the M28 serotype-produced IdeS exhibits a higher affinity for integrin α_V_β_3_, providing an optimized form of IdeS for tumor targeting (71). In fact, CAR-T cell expressing IdeS have been proved to be capable of preventing host humoral immune attack by covering the cell surface using F(ab)_2_ fragments to form a protective layer apart from its anti-phagocytic function (72). To sum up, we conceive that integrating IdeS onto the surface of EVs could potentially achieve both inhibition of phagocytosis as well as specific targeting towards tumor cells, leading to a synergistic anti-tumor effect (**Figure 2B**).

### 5.2 Tumor deactivation via ImpA-mediated cleavage of CD44

CD44, the tumor growth and metastasis-promoting factor, binds various extracellular matrix components, primarily hyaluronic acid (HA) (73). It exists in at least three states: resting, inducible active, and constitutive active. The affinity of CD44 for HA differs based on its activation status. Typically, CD44 maintains the inactive state in normal conditions. When stimulated or located under inflammatory conditions, it turns active along with enhanced HA binding. When expressed by tumor cells, CD44 has constitutive activity, displaying the strongest HA binding ability (74). The abnormally high expression level of CD44 in tumors like pancreatic, colon, osteosarcoma, and lung adenocarcinoma suggests its role in tumorigenesis, invasion, and metastasis. This is supported by the fact that miR-34a which decreases CD44 expression in prostate cancer stem cells can block tumor growth and metastasis (73, 75–77). Therefore, CD44 is a crucial deactivation target for tumor therapy. Considering that the stem region linking extracellular domain of CD44 with its membrane portion may undergo cleavage by membrane-associated metalloproteinases such as MT1-MMP, ADAM10, MMP-9 as well as the aforementioned bacterial protein ImpA from P. aeruginosa, resulting in disruption of the signaling pathways mediated by CD44 and subsequent inhibition of tumorigenesis (78), We conceive that integrating ImpA protein onto the surface of EVs could potentially achieve both inhibition of phagocytosis as well as specific deactivation of tumor cells, leading to a synergistic anti-tumor effect (**Figure 2B**).

## 6 Discussion

Extracellular vesicles (EVs), including exosomes, microvesicles, and apoptotic bodies, work as critical intercellular communication mediators in both physiological and pathological processes. Leveraging their inherent targeting capability, low immunogenicity, and fine biocompatibility, EVs are being widely explored in the treatment of cancer, inflammation, viral infection, and other conditions (1, 3, 7, 79). However, due to the clearance by host mononuclear phagocyte system (MPS), EVs are greatly restrained in therapeutic applications. To overcome this, previous work has utilized host CD47 molecules to engineer EVs and seek to evade phagocytosis through mimicking the tumor evasion mechanism mediated by CD47-SIRPα signaling pathway (27, 80, 81). However, being a host endogenous protein and also a highly expressed protein in tumors, CD47 is involved in the regulation of numerous physiological processes such as cell cycle control, cellular senescence, etc., posing risks of disrupting normal cell fates and promoting tumorigenesis and metastasis (31, 32, 82).

In this study, we propose a novel strategy where bacterial anti-phagocytic proteins are utilized to decorate EVs, mimicking the evasion mechanism of bacteria against host phagocytosis. In fact, bacteria have already evolved quite versatile proteins to counteract host immune system, for example, by binding or cleaving host phagocytosis-related molecules including immunoglobulins, complement molecules, complement receptors, or complement regulatory proteins, or by utilizing polysaccharide capsules on their surface to hinder the recognition of bacterial by hosts (35, 36, 45, 49, 51, 55). Given that pre-loading and post-loading strategy strategies have been developed for EVs, thus, the study discussed here, by incorporating bacterial anti-phagocytic proteins, for the first time systematically expands the pool of candidate molecule candidates qualified for anti-phagocytic surface modification towards EVs. Moreover, since the combination of EVs with two of the candidate proteins, IdeS or ImpA, can not only enhance the drug delivery efficiency but also further improve tumor targeting specificity or promote tumor deactivation, the study here also enables an innovative synergistic EV-based cancer therapy approach.

However, it’s worth noting that careful inspection and further studies are warranted to confirm whether engineered EVs carrying bacterial anti-phagocytic protein candidates possess low immunogenicity, good stability, and excellent safety considering that all these proteins are exogenously derived molecules. As far as is known, IdeS, among others, is the least to worry about since it has been approved by the European Union as a drug for desensitization therapy in highly sensitized transplant patients in 2020 (83). Previous studies have shown that after receiving IdeS at a dosage of 0.25 mg/kg body weight, patients experience rapid and effective degradation of plasma IgG within 24 hours, with IgG level beginning to recover approximately one week later. IdeS can achieve rapid consumption of IgG while do not induce long-term suppression of protective antibodies, demonstrating good safety and tolerability (84). As for other candidates, for example, SpA from S. aureus, assessment of its *in vivo* safety has been conducted in mice, monkeys and human beings respectively, none of which reveals any safety issues (85–87). Therefore, we believe that the combination of bacterial anti-phagocytic proteins with EVs holds considerable potential in enlarging the therapeutic application of EVs, with IdeS further standing out as a most preferred candidate due to its excellent safety and tolerability. Along with future inspection of the immunogenicity and safety of bacterial anti-phagocytic proteins-decorated EVs, AI-aided rational design could be integrated by engineering bacterial proteins to reduce their toxicity and unwanted signal transactivation activities etc. while retaining their ability to inhibit phagocytosis. This shall be particularly helpful for those proteins such as CagA and other toxic anti-phagocytic molecules harboring disease-inducing capabilities.

## Data availability statement

The original contributions presented in the study are included in the article/supplementary material. Further inquiries can be directed to the corresponding author/s.

## Author contributions

JS: Writing – review & editing, Writing – original draft, Software, Investigation, Conceptualization. CcC: Writing – review & editing, Investigation. PP: Writing – review & editing, Investigation. KZ: Writing – review & editing, Investigation. JX: Writing – review & editing, Investigation. CC: Supervision, Project administration, Funding acquisition, Conceptualization, Writing – review & editing, Investigation.
